# Spatiotemporal prediction of mining-induced surface subsidence using integrated D-InSAR and boltzmann time function models

**DOI:** 10.1371/journal.pone.0337631

**Published:** 2026-04-03

**Authors:** Weiwei Zhou, Youfeng Zou, Huabin Chai, Miaomiao Ma, Lailiang Cai, Jibiao Hu

**Affiliations:** 1 Henan Polytechnic University, Jiaozuo, China; 2 College of Geoscience and Surveying Engineering, China University of Mining and Technology (Beijing), Beijing, China; Indian Institute of Technology Jammu, INDIA

## Abstract

With the expansion of underground coal mining, accurate prediction of surface subsidence dynamics has become increasingly critical. This study proposes a hybrid prediction framework integrating Differential Interferometric Synthetic Aperture Radar (D-InSAR) and the Boltzmann time function to address this challenge. In low-gradient deformation zones at the edges of subsidence basins, D-InSAR time-series deformation data are directly coupled with the Boltzmann function to achieve spatiotemporal subsidence prediction. In contrast, for high-gradient central zones where InSAR measurements are affected by decorrelation, edge deformation constraints derived from D-InSAR are combined with leveling observations to invert parameters of the Probability Integral Method (PIM), which are subsequently fused with Boltzmann model parameters to construct a spatiotemporal coupling model. Parameter inversion analysis reveals quantitative relationships between the Boltzmann parameters and key influencing factors, including maximum subsidence, mining rate, and overburden lithology. Validation at the 2301 working face demonstrates that the proposed framework achieves stable dynamic prediction performance, with relative errors ranging from 2.1% to 7.0%, in good agreement with field measurements. The proposed framework enables continuous subsidence prediction across the entire mining basin and provides effective technical support for mining-induced subsidence management and ecological restoration. Overall, the paradigm integrating remote sensing observations, time-dependent functions, and spatial modeling offers a scalable and adaptable solution for dynamic subsidence prediction in similar mining regions.

## 1. Introduction

Coal remains the dominant energy source in China and continues to serve as a cornerstone of the national energy structure [1, 2]. However, intensive underground coal extraction inevitably leads to the redistribution of in-situ stress and the fracturing of overlying strata, which progressively propagates deformation toward the surface. This process often results in surface subsidence, ground fissures, and ecological degradation, posing significant risks to regional environmental stability and public safety [3–6]. Consequently, accurate prediction of the temporal and spatial evolution of mining-induced subsidence is essential for ensuring the safe exploitation of underground resources and guiding the ecological restoration of mining-affected regions.

Traditional ground-based monitoring techniques—such as leveling, triangulation, and GPS surveys—have been widely used for subsidence observation. While these methods provide reliable point-based measurements, they are constrained by high operational costs, limited spatial coverage, and challenges in maintaining long-term monitoring networks. The emergence of Differential Interferometric Synthetic Aperture Radar (D-InSAR) technology has offered an advanced, cost-effective alternative for monitoring mining-induced deformation. D-InSAR enables high-precision, large-scale, and all-weather monitoring of ground displacement, and numerous studies have verified its effectiveness in detecting and quantifying subsidence patterns across mining areas [7–9].

Despite these advantages, D-InSAR still encounters significant limitations in areas experiencing rapid or large-magnitude deformation. Severe decorrelation and phase unwrapping errors in high-gradient subsidence zones often result in missing or unreliable deformation data, particularly near the center of subsidence basins. These data gaps substantially reduce the accuracy and spatial completeness of subsidence prediction [10–12]. To mitigate such issues, researchers have explored coupling InSAR-derived deformation data with analytical time function models to describe the temporal evolution of mining subsidence [13–16]. Classical time function models—such as the exponential, hyperbolic, and Knothe functions—have provided important theoretical insights into subsidence dynamics [17,18]. However, these models often rely on simplified assumptions, exhibit weak physical interpretability, and suffer from complex parameter inversion procedures, which restrict their applicability in dynamic prediction [19].

In response to these limitations, a series of improved time function models have been developed, including the Logistic, Weibull, and Bertalanffy functions [20–21]. Although these models enhance curve-fitting flexibility, each has intrinsic drawbacks. The Logistic model predicts non-zero initial deformation velocity, inconsistent with physical reality; the Weibull model lacks explicit physical meaning; and the Bertalanffy model introduces nonlinear terms that complicate parameter estimation. Moreover, most existing time function models are primarily applied to single-point time-series fitting, failing to achieve full spatiotemporal prediction of deformation across entire subsidence basins [22,23].

To overcome these challenges, this study proposes a hybrid dynamic subsidence prediction framework that integrates D-InSAR observations, the Boltzmann time function, and the Probability Integral Method (PIM). The proposed approach utilizes D-InSAR-derived deformation in marginal low-gradient zones and incorporates leveling observations from central high-gradient regions to invert PIM parameters. These parameters are then coupled with Boltzmann time function parameters to construct a continuous spatiotemporal prediction model. This integration effectively compensates for decorrelation-induced data loss, enhances model completeness, and provides a physically interpretable parameter system linking subsidence evolution to geological and mining factors.

The main objectives of this study are threefold: (1) to develop an integrated D-InSAR–Boltzmann–PIM framework capable of achieving continuous and accurate dynamic subsidence prediction across entire mining basins; (2) to establish quantitative relationships between the parameters of the Boltzmann model and key geological–mining factors, such as maximum subsidence, mining rate, and overburden lithology; and (3) to validate the proposed framework through case studies, assessing its predictive accuracy and applicability for dynamic monitoring and ecological restoration.

The remainder of this paper is organized as follows. Section 2 introduces the study area and dataset. Section 3 details the methodology, including the Boltzmann and PIM models and their integration with D-InSAR data. Section 4 presents the experimental results and validation. Section 5 discusses the findings and limitations, and Section 6 concludes the study.

## 2. Study area and data description

The study area is located at the southern margin of the Qinshui Basin, on the western flank of the southern section of the Taihang Mountains. The regional geomorphology is characterized by erosional mountainous terrain, mainly consisting of low mountains and hilly landscapes dissected by dense gullies and valleys. This study focuses on the 2301 working face, which has a strike length of 996 m, an inclined length of 176 m, an average mining depth of approximately 390 m, a coal seam dip angle of 5°, and a mining thickness of 2.6 m. For field monitoring, a semi-observation line was arranged along the strike direction, and a full observation line was established along the dip direction.

Sentinel-1A data provided by the European Space Agency (ESA) were used in this study, including Single Look Complex (SLC) images acquired in VV polarization and IW (Interferometric Wide swath) mode, with a revisit interval of 12 days. The observation period extended from July 16, 2015, to May 23, 2016, during which a total of 12 Sentinel-1A scenes covering the 2301 working face were collected. Although 12 scenes were available, the dataset was still insufficient for constructing a sufficiently dense and stable SBAS-InSAR interferometric network. Moreover, the monitoring period was relatively short, and mining-induced deformation in the central subsidence zone exhibited strong spatial gradients and severe decorrelation, which could further reduce the reliability and continuity of SBAS-derived time-series results. Therefore, D-InSAR was adopted in this study to extract deformation information for successive monitoring intervals.

For D-InSAR processing, 11 interferometric pairs were generated in chronological order from the 12 available scenes, with temporal baselines of 24 or 48 days and perpendicular baselines ranging from −141 m to 108 m (Table 1). A 30-m-resolution Digital Elevation Model (DEM) was used to remove the topographic phase, and precise orbit ephemeris data were applied to improve orbital accuracy. The interferometric processing mainly included co-registration, interferogram generation, topographic phase removal, adaptive filtering, phase unwrapping, geocoding, and conversion of line-of-sight (LOS) deformation into vertical deformation. Considering that horizontal movement in the study area was much smaller than vertical subsidence, the LOS deformation was projected to the vertical direction for subsequent analysis. Detailed data specifications are summarized in Table 1.

**Table 1:** Parameters of Sentinel-1A SAR image pairs used for D-InSAR processing.

Image pair	Interferogram pair date		temporal baseline/d	vertical baseline/m
	master image	slave image		
1	20150716	20150809	24	−141
2	20150809	20150902	24	69
3	20150902	20150926	24	23
4	20150926	20151020	24	−73
5	20151020	20151113	24	108
6	20151113	20151207	24	−56
7	20151207	20151231	24	−39
8	20151231	20160217	48	32
9	20160217	20160312	24	−6.9
10	20160312	20160405	24	−13.8
11	20160405	20160523	48	59

## 3. Methods

### 3.1. Boltzmann function model construction method

Boltzmann extended the Maxwell distribution to the Maxwell-Boltzmann distribution, and this research achievement has been widely applied in various fields, including aerodynamics and electromagnetism. Later, scholars applied it to mining subsidence prediction [24,25], and the equation expression of the time function is as follows:


$$w\left(t\right)=\frac{-A}{1+e^{(t-t_{\text{0}})/B}}+A$$ (1)

In the equation, *w(t)* represents the surface subsidence amount at time *t*; *t* is the time interval from the start of mining *t*_*o*_ the predicted moment; *A* represents the final subsidence value, *t₀* denotes the time at which the maximum subsidence rate occurs, and *B* reflects the degree of rapidity of the subsidence process, controlling the steepness of the subsidence curve. From the functional relationship, it is known that the subsidence velocity *v(t)* is the first derivative of *w(t)*, and the subsidence acceleration *a(t)* is the second derivative of *w(t)*. The expressions for both are as follows:


$$v\left(t\right)=\frac{A*e^{(t-t_{\text{0}})/B}}{B*{\left[1+e^{(t-t_{\text{0}})/B}\right]}^{\text{2}}}$$ (2)

$$a\left(t\right)=\frac{A*e^{(t-t_{\text{0}})/B}*\left[1-e^{(t-t_{\text{0}})/B}\right]}{B^{\text{2}}*{\left[1+e^{(t-t_{\text{0}})/B}\right]}^{\text{3}}}$$ (3)

### 3.2. PIM construction method

**Fig 1:**
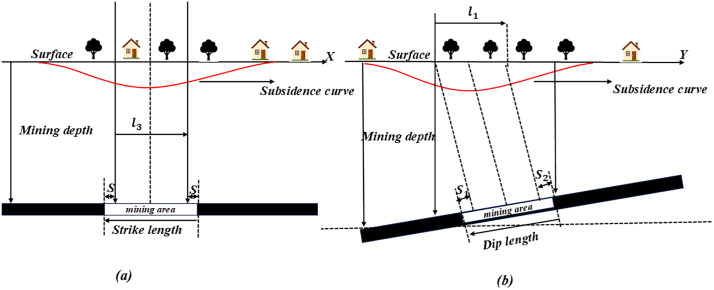
Schematic diagrams of predicted surface movement in (a) the strike section and (b) the dip section based on the PIM.

PIM is a widely used method for predicting coal mining subsidence in China. Fig 1 shows the schematic diagram of subsidence prediction in the strike and dip directions.

The calculation formula for subsidence prediction using the PIM is as follows:


$$\left\{ \begin{array}{c} @l@W_{max}=m*q*cos\;a\\ l=L_{\text{0}}-S_{\text{1}}-S_{\text{2}}\\ L=(L_{\text{1}}-S_{\text{3}}-S_{\text{4}})*\frac{sin(\theta +a)}{sin\;a}\\ W^{\text{0}}\left(x\right)=\frac{W_{max}}{\text{2}}*\left[erf\left(\sqrt{\pi }\frac{x*tan\;\beta }{H_{\text{0}}}\right)-erf\left(\sqrt{\pi }\frac{(x-l)*tan\;\beta }{H_{\text{0}}}\right)\right]\\ W^{\text{0}}\left(y\right)=\frac{W_{max}}{\text{2}}*\left[erf\left(\sqrt{\pi }\frac{y*tan\;\beta }{H_{\text{1}}}\right)-erf\left(\sqrt{\pi }\frac{(y-L)*tan\;\beta }{H_{\text{2}}}\right)\right]\\ W\left(x,y\right)=\frac{\text{1}}{W_{max}}*W^{\text{0}}\left(x\right)*W^{\text{0}}\left(y\right) \end{array}\right.$$ (4)

In the formula, *L* represents the calculated length of the inclined working face. *W*_*max*_ is the maximum surface subsidence value, *m* is the coal seam thickness, *q* is the subsidence coefficient; *a* is the coal seam dip angle, *L*_*0*_ is the coal seam strike length, *L*_*1*_ is the working face dip length, *S*_*1*_ and *S*_*2*_ are the strike left and right turning point offsets, *S*_*3*_ and *S*_*4*_ are the upslope and downslope turning point offsets, *θ* is the main influence propagation angle, *tanβ* is the tangent of the main influence angle, *H*_*0*_ is the average mining depth, *H*_*1*_ is the mining depth in the downslope direction, *H*_*2*_ is the mining depth in the upslope direction. *W⁰(x)* is the subsidence value at the point with the *x*-coordinate on the strike main section, and *W⁰(y)* is the subsidence value at the point with the *y*-coordinate on the dip main section. The existing geological parameters *GP* and the parameters to be predicted *P* are as follows:


$$\left\{ \begin{array}{c} @l@GP=\left[H_{\text{0}},H_{\text{1}},H_{\text{2}},L_{\text{0}},L\right]\\ P=\left[q,tan\beta ,\theta ,S,b\right] \end{array}\right.$$ (5)

The subsidence value *W(x, y)* in the PIM is related to the parameters *GP* and *P* as follows:


W(x,y)=f(GP,P) (6)

### 3.3. D-InSAR-based dynamic subsidence prediction method for mining areas using PIM and boltzmann models

**Fig 2:**
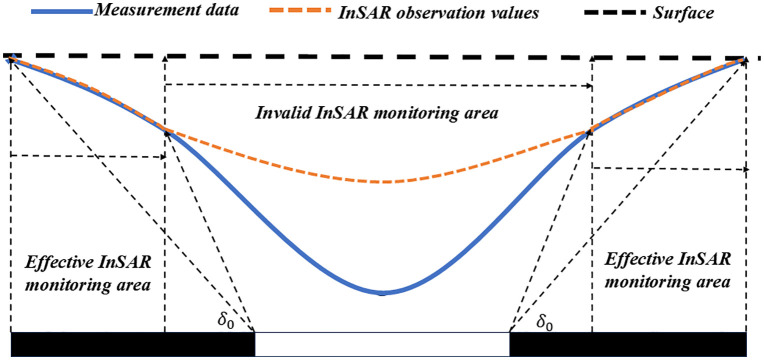
Effective monitoring areas of InSAR for different subsidence zones.

Existing research indicates that InSAR delivers high monitoring accuracy for the edges of subsidence basins, as shown in Fig 2 [26,27]. Meanwhile, the Probability Integral Method (PIM) exhibits fast parameter inversion convergence speed at the edges of subsidence basins [28]. Therefore, for the edge regions of subsidence basins, the parameters of the Boltzmann time function model can be inverted using the Least Squares (LS) fitting method based on D-InSAR data, thereby enabling dynamic prediction for these regions. For large-scale subsidence center regions that cannot be accurately predicted by D-InSAR alone, the PIM parameters can be inverted by combining the edge region deformation data derived from D-InSAR with leveling measurement data near the subsidence center. After integrating the PIM with the key geological parameters of the mining area, the parameter values of the Boltzmann time function can be determined using the overburden lithology coefficient—since this coefficient directly correlates with the temporal evolution characteristics of mining-induced subsidence. This method ultimately realizes dynamic prediction for the entire subsidence area.

**Fig 3:**
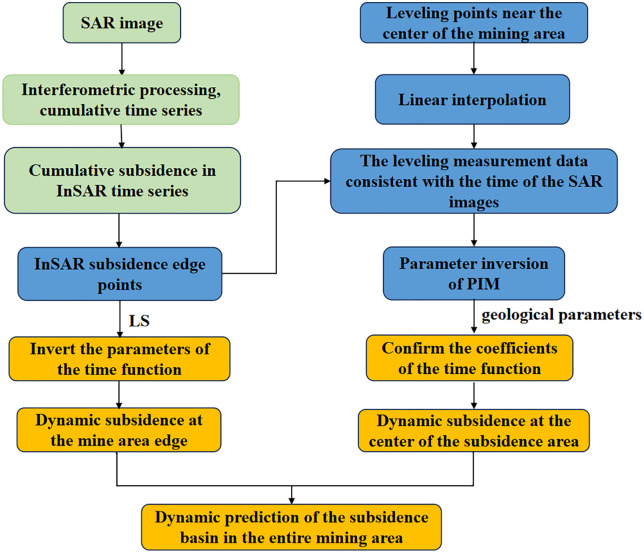
Flowchart of the integrated D-InSAR–PIM–Boltzmann dynamic subsidence prediction model.

Based on the models constructed in Sections 3.1 to 3.3, a process describing the dynamic subsidence basin during the mining process in the mining area can be established, as shown in Fig 3. The specific steps are as follows:

1. D-InSAR Processing: D-InSAR technology is employed to obtain the cumulative line-of-sight deformation of the mined-out area. The line-of-sight deformation is then converted to vertical deformation by considering the radar incidence angle.
2. Inversion of PIM Parameters: The PIM parameters for the mining area are inverted using the LS method, combining the D-InSAR edge results with the measured leveling data near the subsidence center.
3. Complete Mining Area Dynamic Prediction: Dynamic prediction for the mining area’s edge is achieved through the integration of D-InSAR data with the Boltzmann model. For large-scale subsidence centers, the inverted PIM and Boltzmann models are combined, and the time model parameters are determined based on geological parameters and mining conditions. By integrating the generally applicable stable-state PIM method with a dynamic time function, dynamic prediction is realized for the subsidence center, ultimately facilitating dynamic subsidence prediction for the entire mining area.

## 4. Results

**Fig 4:**
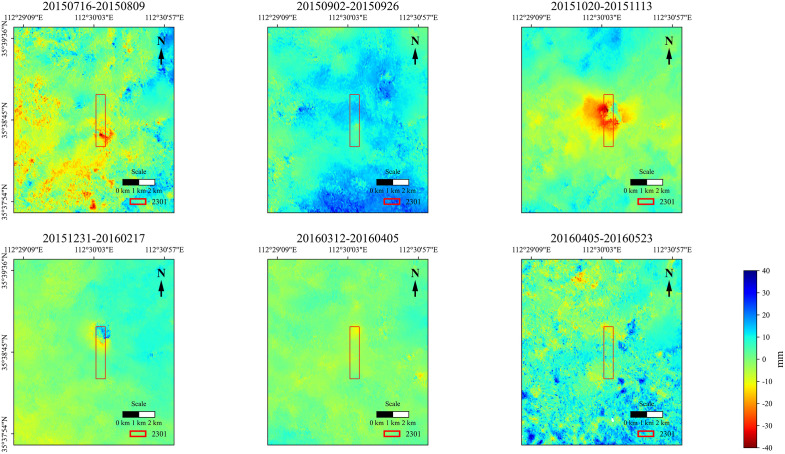
Subsidence magnitudes for each monitoring period derived from D-InSAR processing.

**Fig 5:**
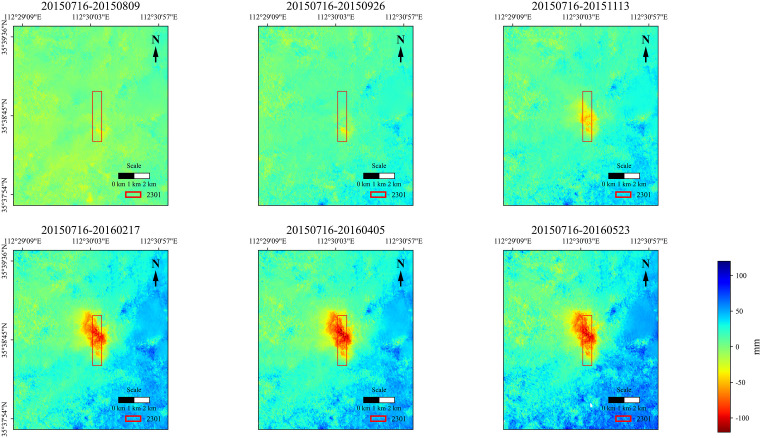
Cumulative subsidence distributions in the 2301 working face over the monitoring period.

The 11 interferometric pairs in Table 1 were subjected to pairwise differential processing in chronological order. Considering that interferometric phases are inevitably affected by various types of noise, an adaptive filtering method was employed for noise removal. The filtered and enhanced differential interferometric phases were further subjected to unwrapping processing. Subsequently, the unwrapped true phases were converted into surface deformation values along the radar line-of-sight (LOS) upward direction. Through projection and geocoding, the true vertical upward deformation values under the geographic coordinate system were obtained. The subsidence amounts during each period and the cumulative subsidence amounts were derived, as illustrated in Fig 4 and Fig 5.

The observation time range of D-InSAR is from July 16, 2015, to May 23, 2016. To save space, only images of 6 periods are presented. From the images of phased subsidence, it can be seen that the subsidence value in the mining area first increases, then decreases with time, and gradually stabilizes. From the images of cumulative subsidence, the subsidence value in the mining area gradually increases over time and finally stabilizes. The maximum monitored subsidence value is 122 mm, which is much smaller than the maximum measured subsidence value.

### 4.1. Probabilistic integral method parameter inversion results

Practical applications have demonstrated that the vertical measurement accuracy of leveling surveys can affect the determination of mining subsidence basin boundaries. To address this issue and optimize the parameter inversion of the PIM, this study employs subsidence values derived from D-InSAR to supplement part of the measured data, thereby reducing the number of leveling points while increasing the number of edge points of the subsidence basin used for parameter inversion. Firstly, edge points of the subsidence basin were selected from the vertical subsidence map obtained by D-InSAR. For leveling points, monitoring points in the mining area center were included. The PIM parameters obtained through joint inversion are summarized in Table 2.

**Table 2:** Inverted parameters of the Probability Integral Method (PIM).

*q*	*θ*	*tanβ*	*S*	*b*
0.72	87.5	2.8	0.1H0	0.25

**Fig 6:**
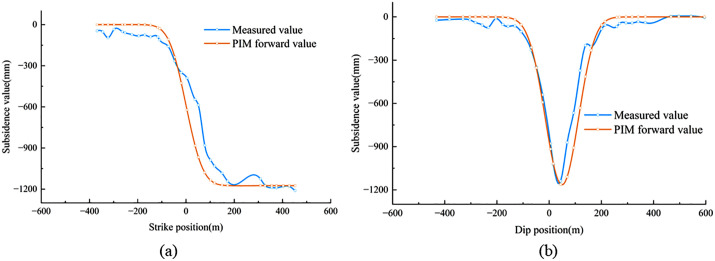
Comparison between measured surface subsidence values and PIM forward-modeled results.

In the formula, *q* represents the subsidence coefficient; *θ* is the mining impact propagation angle; *tanβ* denotes the tangent of the main influence angle; *S* denotes the generalized inflection-point offset parameter, which is represented in the detailed strike and dip formulations by S1–S4 for different directional components; and *b* is the horizontal displacement coefficient. The parameters of the PIM were utilized to forward-model the subsidence values, which were then compared with the measured values, as shown in Fig 6. The mean error and relative error in both the strike and dip directions are summarized in Table 3. From Table 3, it can be observed that the ratios of the mean error and relative error in the strike and dip directions to the maximum subsidence value are 3.8% and 7.0%, respectively, both of which are below 7%. According to relevant theories in the literature [29], the predicted parameters derived from the inverted PIM are considered to be reliable.

**Table 3:** Error statistics between measured and PIM forward-modeled subsidence values in strike and dip directions.

	Maximum Forward Modeling Subsidence (mm)	Mean Error/mm	Relative Error/%
Q	1161	43.59	3.8
Z	1174	89.05	7.0

### 4.2. Complete subsidence dynamic monitoring results of the mining area

By integrating the D-InSAR monitoring results with the Boltzmann model, dynamic subsidence prediction for the edge areas of the mined-out zone can be achieved. Using the parameters inverted through the PIM, as outlined in Section 4.1, the final surface subsidence value in the central region at a stable state can be determined. However, dynamic prediction cannot be realized with this approach alone. This section focuses on combining the PIM with the Boltzmann model to enable dynamic prediction within the central large-scale subsidence area.

#### 4.2.1. Dynamic prediction results of the mining area edge.

To illustrate the fitting performance of the Boltzmann model in the marginal zone of the subsidence basin, three representative points (Points 1–3) were selected from the D-InSAR edge region. These points are located in different parts of the basin edge where deformation gradients are relatively low and D-InSAR observations remain reliable. Their cumulative subsidence magnitudes fall within the low-to-moderate subsidence range of the edge zone. The differences between the cumulative D-InSAR subsidence time series and the corresponding Boltzmann fitting results are summarized in Table 4. The results indicate that the Boltzmann model provides a good fit to the D-InSAR observations in the basin-edge region.

**Table 4:** Boltzmann time-function inversion results for selected points in the D-InSAR edge region.

	*A*	*t*_*0*_	*B*	Mean Error/mm	Relative Error/%
1	20.35	94.98	32.94	1.44	7.0
2	17.35	106.16	45.94	1.29	7.2
3	17.48	133.82	36.73	1.04	5.6

#### 4.2.2. Dynamic monitoring results of the large-scale subsidence center.

1 Determine the value of parameter *A*

To gain a deeper understanding of the physical significance and determination methods of the parameters in the Boltzmann time function model, this study employed the measured data of all monitoring points on the 2301 working face to invert the model parameters and analyze their variation patterns. Following the completion of parameter inversion for all monitoring points, 20 representative monitoring points were selected for more detailed analysis. The inversion results of these monitoring points are summarized in Table 5.

**Table 5:** Boltzmann function parameter inversion results for representative monitoring points in the 2301 working face.

Point	*A*	*t*_*0*_	*B*	Subsidence value calculated by the PIM	Measured Final Subsidence Value/mm	Relative Error between Parameter A and the Subsidence by PIM	Relative Error between Parameter A and the Measured Final Subsidence Value/%
Q013	499.7	86.1	25.2	516	540	3.2	7.4
Q014	829.7	81.1	19.4	843	879	1.6	6.1
Q015	970.3	82.9	20.0	986	1023	1.6	5.1
Q016	1090.4	81.28	19.0	1156	1139	5.7	4.2
Q017	834.8	87.6	20.4	800	870	4.4	4.0
Q018	640.5	88.7	22.1	620	666	3.3	3.8
Q019	364.3	87.4	22.8	395	372	7.8	2.0
Q020	189.2	85.8	21.7	175	197	8.1	3.9
Z016	407.1	19.6	9.0	444	431	8.3	5.5
Z017	620.5	20.7	8.5	675	681	8.0	8.8
Z018	863.1	20.9	6.6	912	918	5.4	5.9
Z019	985.8	21.6	5.7	1086	1032	9.2	4.4
Z020	1040.9	25.8	5.7	1112	1084	6.4	3.9
Z029	1078.0	46.4	9.0	1196	1125	9.8	4.1
Z030	1013.2	52.7	10.5	1112	1058	8.8	4.2
Z031	1109.8	62.4	11.6	1176	1189	5.6	6.6
Z032	963.5	72.7	14.4	1020	995	5.5	3.1
Z033	1022.1	92.8	23.5	1113	1080	8.1	5.3
Z034	1059.7	106.8	24.9	1078	1108	1.7	4.3
Z035	1190.6	123.0	18.2	1231	1211	3.2	1.6

As shown in Table 5, the differences between the Boltzmann parameter *A*, the final subsidence values derived from the PIM, and the measured final subsidence values are relatively small. This result quantitatively verifies that, under the geological and mining conditions considered in this study, approximating parameter *A* as the final subsidence value W(x,y)at surface observation points is reasonable.

2 Determine the value of parameter *t*_*0*_

**Fig 7:**
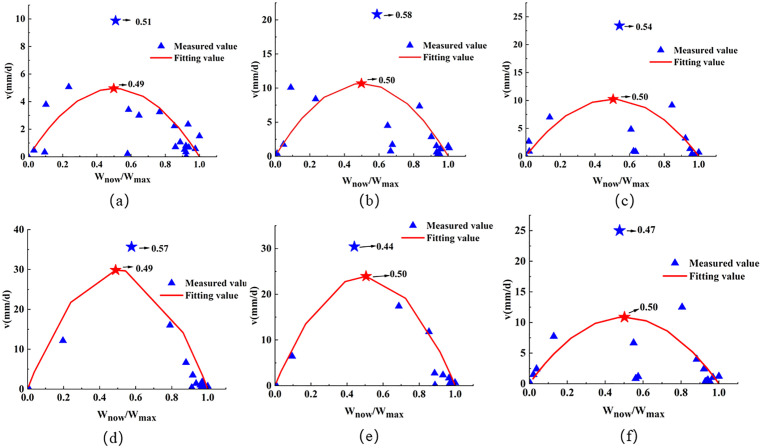
Relationship between subsidence value and maximum subsidence at the time of peak subsidence velocity for representative points in the 2301 working face.

Field measurements indicate that when the subsidence reaches half of its final value, the point experiences the maximum subsidence rate [30,31]. This was verified using the measured data from the 2301 working face: selected monitoring points were used to calculate the ratio of the difference in subsidence values between two time periods to the time interval. Since it is not possible to directly obtain the subsidence rate at any given moment, the Boltzmann time function and the parameters from Table 5 were used to compute the subsidence rate at arbitrary time points. Then, the ratio of the subsidence at the moment of maximum velocity to the final subsidence value was calculated and statistically analyzed. The results are shown in Fig 7. *W*_*now*_ represents the subsidence value at the moment when the maximum velocity occurs, and *W*_*max*_ represents the final subsidence value.

Based on the patterns observed in the measured data, it is assumed that when a surface point reaches its maximum subsidence rate, the mining distance is *s*, and the subsidence at this point is approximately half of the final subsidence value. Therefore, the time *t*_*0*_ at which the maximum subsidence rate occurs can be determined using the following formula:


$$W_{s}\left(x,y\right)=\frac{\text{1}}{\text{2}}W\left(x,y\right)$$ (7)

$$\frac{\text{1}}{W_{max}}*{W_{s}}^{\text{0}}\left(x\right)*W^{\text{0}}\left(y\right)=\frac{\text{1}}{\text{2}}*\frac{\text{1}}{W_{max}}*W^{\text{0}}\left(x\right)*W^{\text{0}}\left(y\right)$$ (8)

$$\begin{array}{c} \frac{W_{max}}{\text{2}}*\left[erf\sqrt{\pi }\frac{x*tan\;\beta }{H_{\text{0}}}-erf\sqrt{\pi }\frac{(x-s\prime )*tan\;\beta }{H_{\text{0}}}\right]\\ =\frac{\text{1}}{\text{2}}*\frac{W_{max}}{\text{2}}*\left[erf\sqrt{\pi }\frac{x*tan\;\beta }{H_{\text{0}}}-erf\sqrt{\pi }\frac{(x-l)*tan\;\beta }{H_{\text{0}}}\right] \end{array}$$ (9)

The above equation can be simplified as:


$$erf\left(\sqrt{\pi }\;\frac{(x-s\prime )*tan\;\beta }{H_{\text{0}}}\right)=\frac{\text{1}}{\text{2}}\left[erf\left(\sqrt{\pi }\;\frac{x*tan\;\beta }{H_{\text{0}}}\right)+erf\left(\sqrt{\pi }\;\frac{(x-l)*tan\;\beta }{H_{\text{0}}}\right)\right]$$ (10)

*W*_*s*_*(x, y)* represents the subsidence value of the monitoring point when mining to point *s*, *W*_*s*_^*0*^*(x)* represents the subsidence value of the monitoring point with the abscissa *x* on the strike main section when mining to point *s*, and the offset distance of the right inflection point *S*_*s2*_ is defined by the ratio of the mining distance to the strike length *l*, that is, *S*_*s2*_=(*s/l*)*S_*2*_. Therefore, the mined distance $s^{\prime }$=*s*-*S*_*1*_-(s/l) * S_*2*_. According to Equation 10, the mining distance *s* at which the maximum speed occurs can be obtained, and the time at which the maximum speed occurs can be calculated according to Equation *t*_*0*_ *= s/c*, where *c* is the mining speed of the working face. In addition, *t*he calculation method is based on the PIM, and the results rely only on theoretical derivation, which may lead to a mining distance *s* that is less than the starting distance of the working face. In practice, if the calculated mining distance *s* is less than the starting distance, it should be considered that the surface has not been significantly deformed, and the mining distance should be recognized as the starting distance. In this case, the calculation formula for *t*_*0*_ should be improved as follows:


$$t_{\text{0}}=\left\{ \begin{array}{c} @l@\frac{s}{c},s>d_{\text{0}}\\ \frac{d_{\text{0}}}{c},s<=d_{\text{0}} \end{array}\right.$$ (11)

where *d*_*0*_ represents the starting distance.

**Fig 8:**
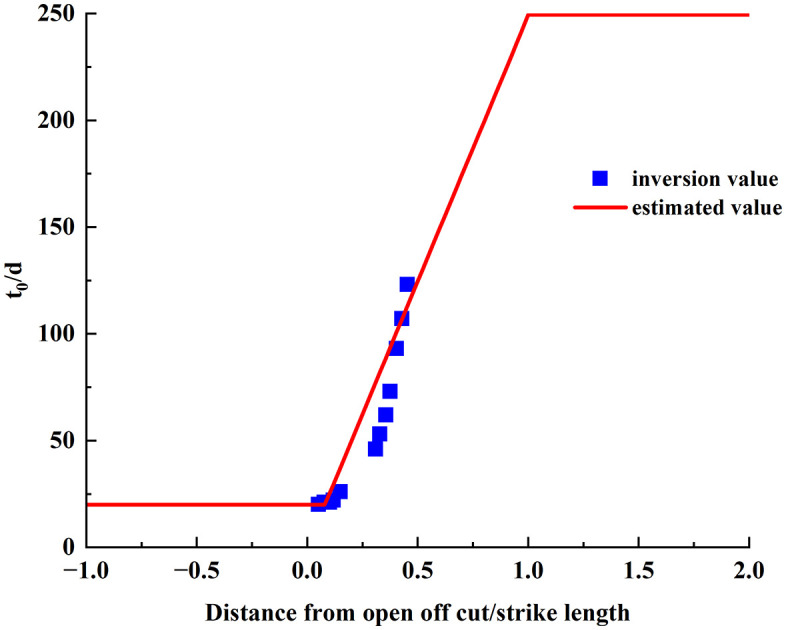
Comparison between inverted and predicted values of the time of maximum subsidence rate (*t₀*) for monitoring points in the 2301 working face.

To verify the reliability of the method, the Boltzmann time function model parameters obtained from the inversion of measured subsidence values in the 2301 working face were used to calculate the time *t*_*0*_ at which the maximum subsidence rate occurs. This calculated *t*_*0*_ was then compared with the predicted *t*_*0*_ from Equation (11). As shown in Fig 8, the time *t*_*0*_ obtained using this method follows the overall time trend well, indicating that the method is reliable.

3 Determine the value of parameter *B*

According to Equation 4, when *t = t*_*0*_, the equation can be written as:


$$B=\frac{A}{4v_{max}\left(x,y\right)}$$ (12)

where *v*_*max*_*(x, y)* represents the maximum sinking velocity at any point. Therefore, the maximum subsidence velocity at any point can be used to calculate the coefficient of subsidence urgency, *B*. To investigate the maximum subsidence velocity at any point in the study area, reference is made to the empirical method commonly used for the overall maximum subsidence velocity of the mine:


$$v_{max}=\frac{p*c*W_{max}}{H_{\text{0}}}$$ (13)

Further extending the derivation, it can be concluded that for any mining area, the maximum subsidence velocity at any surface point is linearly related to the final subsidence value. The corresponding relationship is expressed as follows:


$$v_{max}\left(x,y\right)=\frac{p*c*W\left(x,y\right)}{H_{\text{0}}}$$ (14)

where *p* is the lithological coefficient of overlying rock.

**Fig 9:**
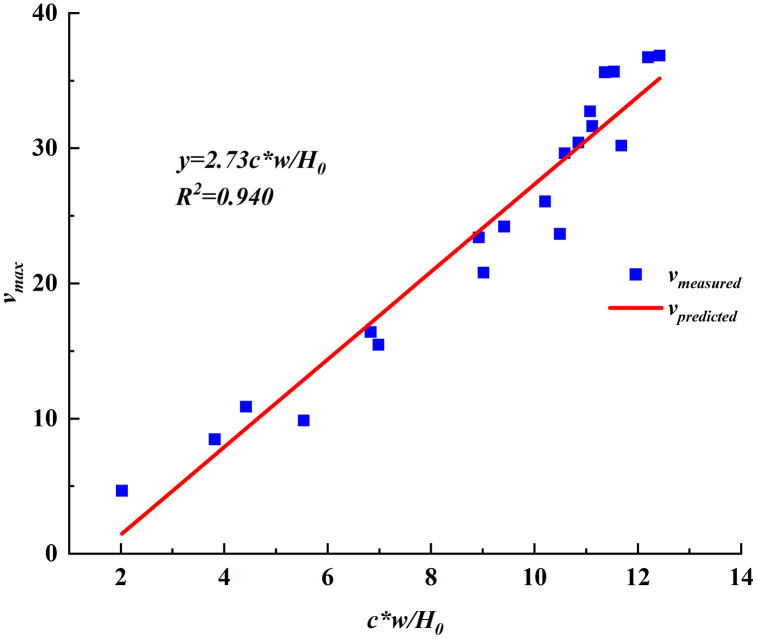
Relationship between maximum subsidence velocity and final subsidence for monitoring points in the 2301 working face.


As shown in Fig 9, the maximum subsidence velocity at any point on the mine surface and the final subsidence value at that point were consistent with this trend. According to the relationship between the maximum subsidence velocity and the final subsidence value, the overburden rock lithology coefficient *p* was fitted, and the value of *p* for this working face was 2.73, that is, the maximum subsidence velocity at any point of the 2301 working face *v*_*max*_*(x, y)*=2.73c*w/*H*_*0*_. When solving the coefficient of the degree of urgency of subsidence for any point *B*, because *A* is identified as the final subsidence value *W(x, y)*, after derivation, we finally obtain *B* as a fixed value with the following expression:

$$B=\frac{H_{\text{0}}}{4p*c}$$ (15)

After introducing the value of *p*, a value of 8.92 was calculated for the 2301 working face for *B*. When inverting the parameters of the Boltzmann time function model using the measured subsidence value data, the coefficient of sinking rapidity *B* varies from point to point because a more accurate prediction can be achieved from a single point. In-depth analyses revealed that treating *B* as a constant had little effect on the maximum subsidence rate at any point on the surface. In addition, treating *B* as a fixed value simplifies the operation of predicting the subsidence dynamics at any point in the mine.

Parameter *A* represents the final subsidence value, which is approximately equal to the final subsidence value at the prediction point and can be determined using the PIM. *t*_*0*_ represents the time at which the maximum subsidence velocity occurs, which is obtained from the PIM. The subsidence value at the prediction point, based on the time of maximum velocity, is derived from the fact that it is half of the final subsidence value. Coefficient *B* represents the degree of rapid or slow subsidence, and its determination is based on the relationship between the maximum subsidence velocity and the final subsidence value at each surface point. This is obtained through fitting the overburden lithology coefficient *p* and combined with geological and mining conditions. Finally, the subsidence model at any point and any time on the surface can be expressed as follows.


$$w\left(x,y,t\right)=\frac{W\left(x,y\right)}{1+e^{4p*c(t-t_{\text{0}})/H_{\text{0}}}}-W\left(x,y\right)$$ (16)

**Fig 10:**
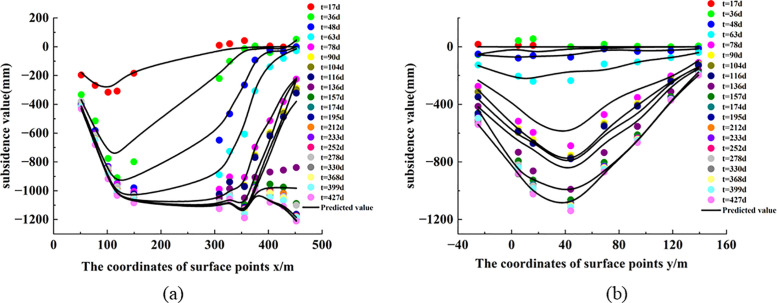
Comparison between measured and predicted subsidence values in the 2301 working face: (a) strike direction; (b) inclined direction.

**Fig 11:**
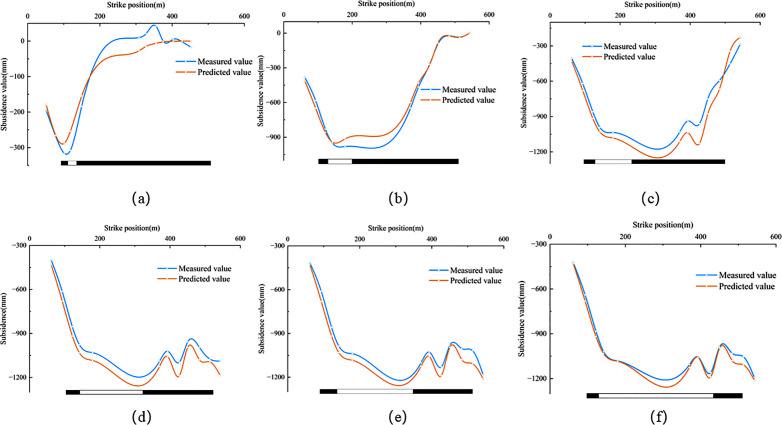
Comparison between predicted and measured subsidence values at strike monitoring points (t = 17d,t = 48d,t = 90d,t = 157d,t = 212d,t = 330d).

**Fig 12:**
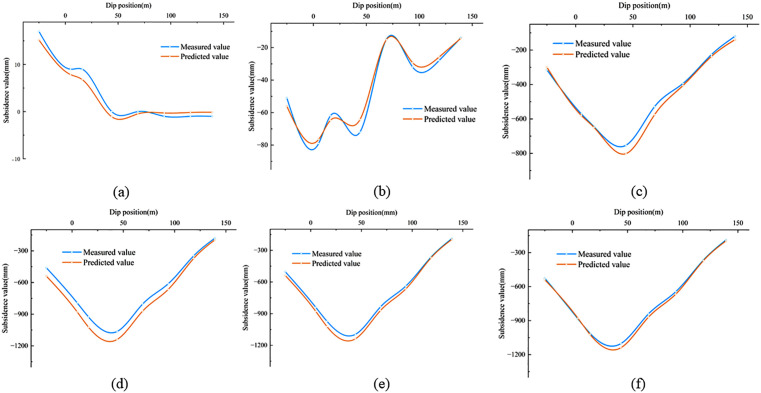
Comparison between predicted and measured subsidence values at dip monitoring points (t = 17d,t = 48d,t = 90d,t = 157d,t = 212d,t = 330d).

In Equation 16, w(*x, y, t)* represents the subsidence value at any time under the coordinates *x* and *y*. According to Equation 16, the subsidence value at any point and at any time in the subsidence center can be calculated by combining the inverted probability integral method with geological and mining conditions. Using the method described above, the subsidence values for all monitoring points in the 2301 working face during the monitoring period were calculated and compared with the measured data. The results are shown in Fig 10. Six periods with significant subsidence changes in the working face were selected for accuracy verification, and the results are presented in Table 6. The results show that the maximum mean error of the dynamic prediction is 72.5 mm, and the minimum is 18.9 mm. The relative error for each period ranges from a maximum of 7.0% to a minimum of 2.1%, with the relative error consistently staying within 7.0%. Therefore, this demonstrates that the method has high accuracy and reliable results. The temporal variation of the errors in Table 6 is closely associated with the evolutionary stage of mining-induced subsidence. At the initial stage, the predicted subsidence magnitude is relatively small, so moderate absolute deviations may still produce a relatively noticeable relative error. During the middle mining stages, the working face is in the rapid subsidence development phase, and both the deformation gradient and subsidence velocity are relatively large. Under such conditions, the dynamic prediction is more sensitive to the inversion accuracy of the Boltzmann parameters, resulting in comparatively larger relative errors. In contrast, during the later stages, the subsidence basin gradually stabilizes and the time-function curve tends toward convergence, so the prediction errors decrease correspondingly. Figs 11 and 12 display the measured and predicted values for the six periods in both the strike and dip directions, showing a good consistency between the measured values and the fitted values. Ultimately, dynamic monitoring of the subsidence center was achieved.

**Table 6:** Accuracy analysis of predicted subsidence values for different monitoring periods.

Mining Time t/d	Predicted Maximum Subsidence Value for This Period/mm	Mean Error/mm	Relative Error/%
17	287.7	18.9	6.0
48	942.3	26.9	2.7
90	1112.3	72.5	7.0
157	1180.0	72.2	6.5
212	1210.0	45.6	3.8
330	1211.0	25.6	2.1

## 5. Discussion

### 5.1. Theoretical constraints and practical limits of D-InSAR in edge subsidence monitoring

The results of this study demonstrate that D-InSAR provides reliable deformation information in marginal zones of subsidence basins where deformation gradients are relatively low. However, its performance degrades significantly toward the central subsidence zone, where large deformation gradients often lead to severe decorrelation and deformation data loss. To clarify the underlying cause of this limitation, the theoretical and practical constraints of deformation gradients detectable by InSAR are analyzed.

After eliminating various interference factors, surface deformation is represented in interferograms as a series of interferometric fringes. It should be noted that the phase information contained in these fringes does not represent absolute deformation but rather the relative phase differences between adjacent pixels. Under ideal conditions, the maximum deformation gradient theoretically detectable by InSAR can be expressed as:


$${\text{d}}_{max}=\lambda /2\mu$$ (17)

Where *d*_*max*_ is the maximum deformation gradient of InSAR theory; *μ* is the pixel size; *λ* is the radar wavelength. However, in practice, InSAR is often affected by geometric incoherence, temporal incoherence, and radar thermal noise. the maximum deformation gradient is smaller than the theoretical value. The maximum deformation gradient that InSAR can actually monitor is:


$$D_{max}=d_{max}+0.002(\gamma -1)$$ (18)

Where *D*_*max*_ is the maximum deformation gradient of the actual InSAR; *γ* is coherence. Finally, the maximum subsidence value *d*_*theory*_ that can be monitored by *N* interference pairs is obtained.


$$d_{theory}=\mu D_{max}N$$ (19)

In this study, the average coherence of the interferometric pairs is approximately 0.5. Given the spatial resolution of Sentinel-1A data (20 m × 20 m) and a radar wavelength of approximately 5.6 cm, the maximum deformation that can be reliably detected is estimated to be about 88 mm. This deformation gradient constraint constitutes a fundamental cause of severe decorrelation and deformation data loss in the central subsidence zone. Consequently, the proposed D-InSAR–Boltzmann–PIM fusion framework is not only technically justified but also practically necessary to achieve continuous and reliable spatiotemporal subsidence prediction across the entire mining basin.

### 5.2. Engineering and practical implications

From an engineering perspective, the proposed D-InSAR–Boltzmann–PIM framework provides a practical tool for dynamic subsidence management in underground coal mining areas. By integrating InSAR-derived deformation in low-gradient zones with leveling observations in high-gradient regions, the framework enables continuous spatiotemporal prediction of surface subsidence across entire mining basins. This capability is particularly valuable for identifying high-risk subsidence zones, supporting early warning of ground fissures, and guiding the protection of surface infrastructure such as buildings, roads, and pipelines.

In addition, the physically interpretable parameters of the Boltzmann time function offer meaningful insights into the temporal evolution of subsidence, allowing mining engineers to assess the effects of mining rate, extraction intensity, and overburden conditions on subsidence dynamics. This information can be used to optimize mining parameters and adjust extraction schedules to mitigate surface damage. Furthermore, the predicted subsidence evolution can support post-mining ecological restoration by informing land reclamation planning, vegetation recovery assessment, and long-term stability evaluation of mining-affected areas. Overall, the proposed framework bridges the gap between remote sensing–based monitoring and engineering-oriented subsidence control, providing a practical reference for sustainable mining management and ecological restoration.

Compared with the traditional PIM method, which can only predict the final subsidence value, this paper realizes the dynamic subsidence prediction of the complete mining area by combining the time function and PIM method with D-InSAR technology. However, there is a lack of systematic comparison of the existing dynamic subsidence prediction technology, such as with the deep learning method, which will be further studied in the follow-up study.

### 5.3. Sensitivity analysis of parameter *B*

To evaluate the computational robustness of treating the Boltzmann parameter *B* as a constant for the 2301 working face, a sensitivity analysis was conducted by perturbing *B* within ±10% and ±20% of its baseline value (8.92), while keeping all other parameters unchanged. Table 7 summarizes the corresponding prediction errors at the mining time of 212 days. The results indicate that moderate variations in *B* mainly affect the rate of subsidence evolution, whereas the overall prediction accuracy remains relatively stable within the tested range. As the deviation of *B* from the baseline value increases, the prediction errors gradually increase, with more pronounced effects observed during the acceleration and deceleration stages of subsidence. These findings suggest that the assumption of a constant *B* is reasonable under approximately homogeneous overburden conditions, while adaptive adjustment of *B* is required in mining areas characterized by heterogeneous lithology.

**Table 7:** Sensitivity analysis of prediction accuracy with respect to parameter *B.*

*B*	Variation	Mean Error/mm	Relative Error/%
7.14	−20%	80.4	6.7
8.03	−10%	64.8	5.4
8.92	0	45.6	3.8
9.81	10%	68.4	5.7
10.7	20%	84	7.0

In mining areas characterized by heterogeneous overburden lithology, the subsidence rapidity may vary spatially due to differences in mechanical properties and stratigraphic structures. To address this issue, parameter *B* in the Boltzmann time function can be extended from a constant value to a zonally adaptive parameter. Specifically, the mining area can be partitioned into several lithological or structural zones based on overburden composition, key strata distribution, or geological logging information. Within each zone, a representative lithology coefficient *pᵢ* is assigned, and the corresponding Boltzmann parameter *Bᵢ* is determined accordingly.

The zone-specific *Bᵢ* values can be calibrated using a limited number of leveling observations or reliable InSAR-derived deformation time series within each zone, while other parameters remain unchanged. To ensure physical continuity and avoid abrupt parameter jumps at zone boundaries, spatial smoothing or continuity constraints can be imposed during calibration. This zonal adaptive strategy allows the proposed framework to better capture spatial variability in subsidence dynamics while maintaining model stability and computational efficiency. The detailed implementation and validation of this adaptive *B* scheme will be explored in future work.

## 6. Conclusions

This study proposes a spatiotemporal coupling prediction framework for mining-induced subsidence by integrating the Probability Integral Method (PIM) with the Boltzmann time function, aiming to overcome the coverage limitations of D-InSAR in high-gradient deformation zones. By combining remote sensing observations with physical and time-dependent models, the framework enables full-basin dynamic subsidence modeling from marginal areas to central subsidence zones. In low-gradient regions, D-InSAR-derived time-series deformation data are effectively coupled with the Boltzmann model to achieve dynamic subsidence prediction, while in central regions affected by decorrelation, a joint inversion strategy integrating D-InSAR edge constraints and leveling observations is employed to derive reliable PIM parameters. The fused PIM–Boltzmann framework achieves continuous spatiotemporal prediction across the entire subsidence basin, with validation at the 2301 working face indicating dynamic relative errors within the range of 2.1–7.0%.

The main contributions of this study can be summarized as follows. First, a physically interpretable and data-fusion-based dynamic subsidence prediction framework is established, effectively bridging remote sensing observations and classical physical modeling. Second, the coupling mechanism between PIM and the Boltzmann time function provides a practical and technically justified solution for mitigating deformation data loss in high-gradient subsidence zones, thereby improving the completeness and reliability of InSAR-based deformation analysis. Third, the proposed framework enhances the capability for large-scale and continuous dynamic subsidence prediction, offering robust technical support for mining subsidence management and ecological restoration planning.

Despite its demonstrated effectiveness, several limitations remain. The proposed framework currently assumes relatively homogeneous geological conditions within the prediction domain and relies on leveling data for parameter calibration in central subsidence zones. Future research will focus on incorporating multi-source deformation datasets, such as SBAS-InSAR, UAV photogrammetry, and three-dimensional geological models, to improve parameter estimation under heterogeneous geological conditions. In addition, the integration of machine learning or data assimilation techniques may further enhance model adaptability and prediction performance. Although the present validation is conducted at the 2301 working face, the proposed framework is designed to be adaptable to different geological and mining conditions through site-specific parameter calibration, which will be further examined through multi-site applications in future work.

Overall, this study provides a scalable and physically grounded approach for dynamic subsidence prediction in mining areas. The paradigm integrating remote sensing observations, time-dependent functions, and spatial modeling offers a valuable reference for advancing intelligent deformation monitoring and sustainable ecological restoration in mining-affected environments.
